# ST Elevation in aVR: An Atypical Presentation of Pulmonary Embolism

**DOI:** 10.1002/ccr3.70671

**Published:** 2025-07-29

**Authors:** Bernard R. Francis, Nouman Arshad, Mohammad El‐Din, Ibrahim Antoun

**Affiliations:** ^1^ Department of Cardiology Kettering General Hospital Kettering UK; ^2^ Department of Cardiovascular Sciences University of Leicester Leicester UK

**Keywords:** aVR, coronary angiogram, electrocardiogram, pulmonary embolism, ST elevation

## Abstract

ST elevation in aVR on the electrocardiogram (ECG) indicates high‐risk acute coronary syndrome (ACS) but is rarely reported in pulmonary embolisms (PEs). We present a 47‐year‐old female with a history of PE and ankylosing spondylitis admitted for chest pain, nausea, and an episode of possible collapse. Her ECG showed ST elevation in aVR with anterolateral ST depressions, prompting a normal emergency coronary angiogram. Bedside echocardiography revealed right ventricular (RV) dilatation, and lab tests showed elevated D‐dimer levels and troponin. Urgent computed tomography of the pulmonary arteries (CTPA) confirmed large bilateral PEs. The patient was treated with Enoxaparin and transitioned to Warfarin, resulting in symptom improvement. ST‐segment elevation in lead aVR may mimic ACS but suggests significant conditions like PE, often from RV strain and impaired coronary blood flow due to acute RV failure. Clinicians should suspect PE in patients with aVR changes, especially with relevant clinical history and signs of RV pressure overload on echocardiography, to prevent misdiagnosis and ensure timely care.

## 1 Introduction

Cardiovascular disease is becoming a healthcare challenge, especially in the developing world [1, 2, 3]. Pulmonary embolism (PE) is a potentially life‐threatening condition with diverse clinical and electrocardiographic presentations. Among these, ST‐segment elevation in lead aVR is a rare but noteworthy finding that can mimic acute coronary syndromes (ACS), particularly left main or triple‐vessel coronary disease [4, 5]. This can lead to diagnostic confusion and inappropriate intervention. One proposed mechanism for aVR elevation in PE is acute RV strain, which causes subendocardial ischemia due to increased afterload. Bedside echocardiography plays a critical role in differentiating PE from ACS, especially when the ECG findings are ambiguous. This case highlights the importance of recognizing ECG patterns in conjunction with clinical and imaging findings to avoid misdiagnosis and ensure timely management.

## 2 Case History and Examination

**FIGURE 1:**
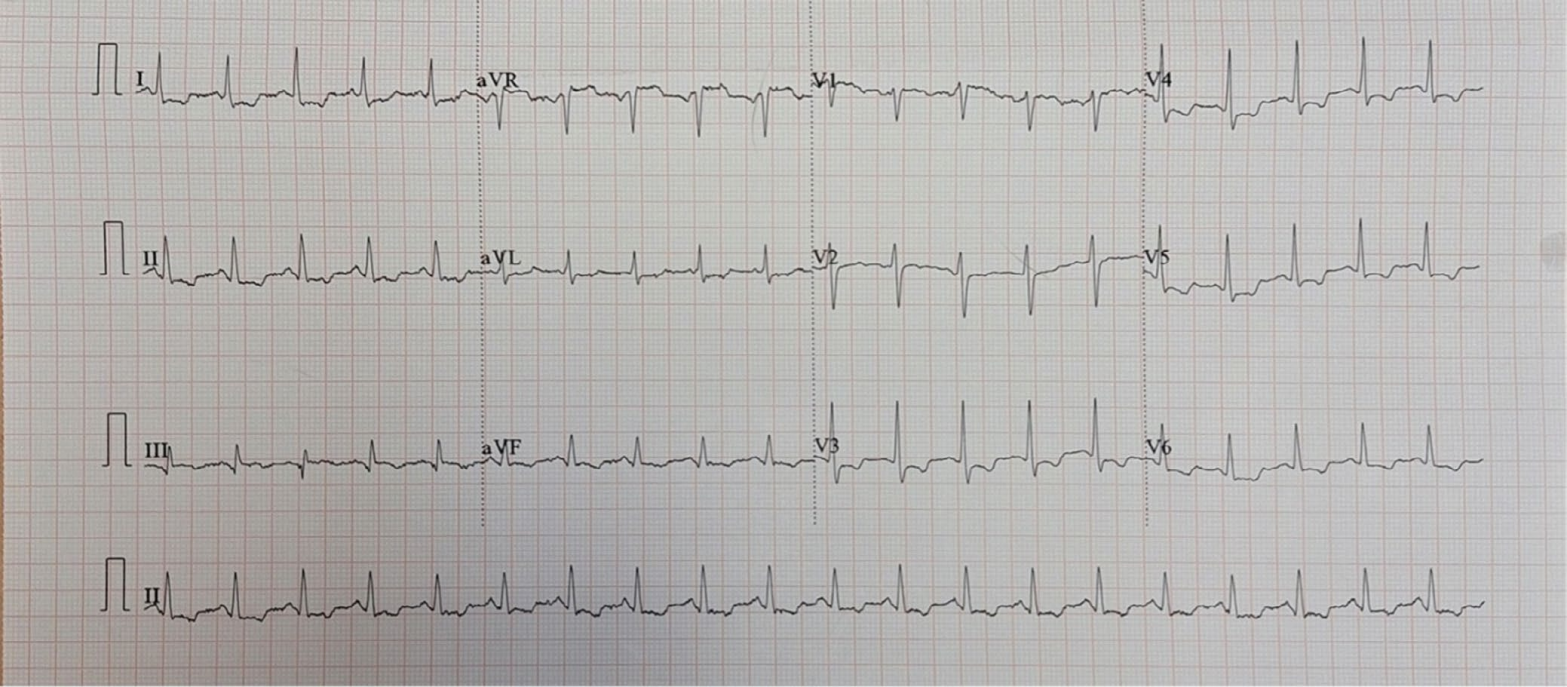
Twelve‐lead electrocardiogram on admission demonstrating ST elevation in aVR with anterolateral ST depressions.

Our patient is a 47‐year‐old female who presented to the hospital due to intermittent central chest pain for the past 3 h. The patient could not give a detailed history of the pain's characteristics. She had a PE 3 years ago and has been on Apixaban 5 mg twice a day (bd). She was having a flare of ankylosing spondylitis and was unable to get out of bed over the last week before admission. The patient self‐discontinued her anticoagulation without medical consultation, citing nausea and difficulty with oral intake. Upon presentation, she developed further chest pain with radiation to her shoulders, nausea, and vomiting. She also had an episode of possible collapse at home. Her physical examination was insignificant. Her ECG showed ST elevation in aVR with anterolateral ST depressions (Figure 1). On admission, her heart rate was 140 beats per minute, her blood pressure was 150/95 mmHg, and her respiration rate was 30 breaths per minute. Her oxygen saturations were 98% on 2 L of oxygen.

## 3 Differential Diagnosis and Diagnostic Tests

**FIGURE 2:**
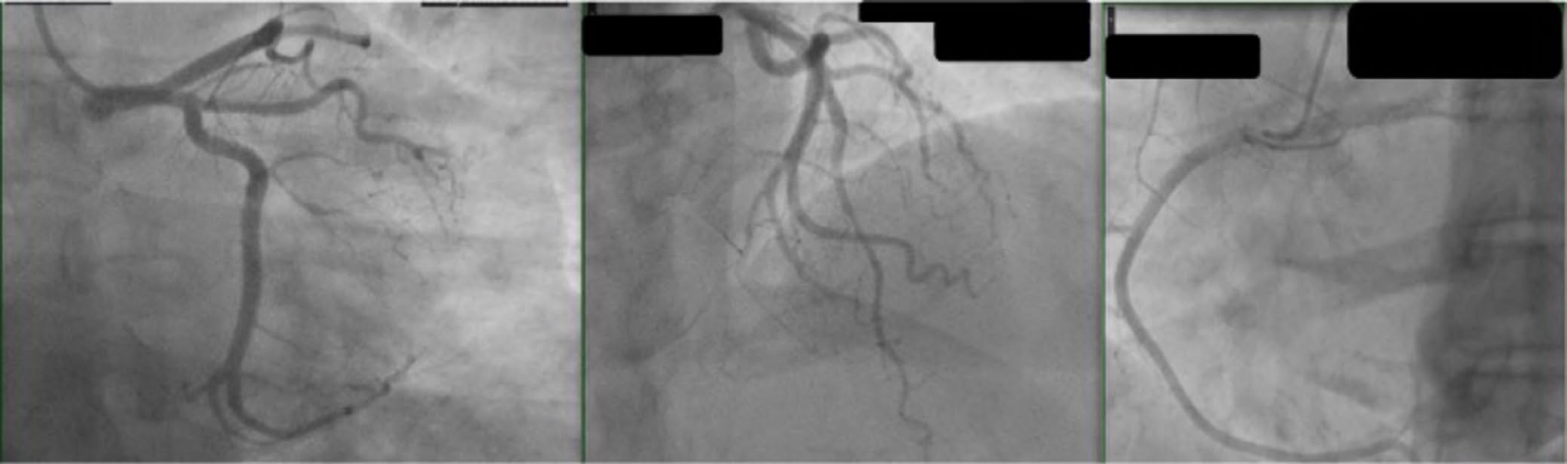
Invasive coronary angiogram ruling out flow‐limiting lesions.

**FIGURE 3:**
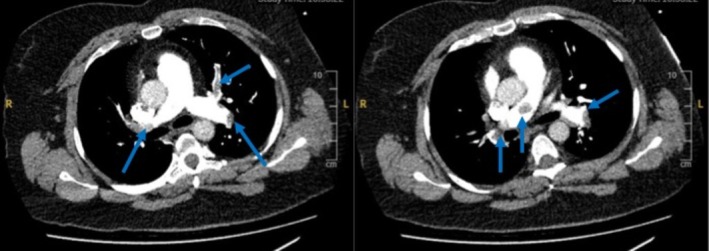
Computed tomography scan of the pulmonary artery showing extensive bilateral thromboembolism in the main and branch pulmonary arteries (blue arrows).

Although a formal clinical probability score was not calculated at the time, the patient's presentation fulfilled multiple criteria of the Wells score. Retrospective calculation yielded a total score of 9.5, indicating high clinical probability. The criteria included previous PE (1.5 points), heart rate > 100 bpm (1.5 points), recent immobilization (1.5 points), hemoptysis (not present), and no alternative diagnosis more likely than PE (3 points). The blood tests were not back at that time. However, a venous blood gas showed a lactate of 5.2 mmol/L and a pH of 7.32. In the context of this ECG, the ST elevation myocardial infarction (STEMI) pathway was activated, and she was taken to the catheter laboratory for an emergency coronary angiogram, which was normal (Figure 2). A bedside echocardiogram performed on the catheter laboratory table revealed marked right ventricular dilatation with a D‐shaped septum, visually impaired systolic function, and an RV‐to‐LV ratio approaching 2. Quantitative measures revealed a TAPSE of 13 mm (normal > 17 mm), indicating impaired RV longitudinal function, and an estimated RV systolic pressure (RVSP) of 50 mmHg, consistent with moderate pulmonary hypertension. Her RV‐to‐left ventricle ratio was almost 2. Lab results showed D‐dimers greater than 10,000 ng/mL (reference: < 500 ng/mL) and a troponin level of 177 ng/dL (reference: 0–12 ng/dL). Although a formal clinical probability score was not calculated at the time, the patient's presentation retrospectively satisfies criteria for a high‐probability Wells score (score > 6) due to recent immobilization, prior PE history, tachycardia, and clinical signs suggestive of PE. Subsequently, she had an urgent computed tomography of the pulmonary arteries (CTPA), which showed a large burden of bilateral PEs in the main pulmonary arteries (Figure 3). There was no indication of thrombolysis at the time, and she only required 2 L of oxygen through a nasal cannula, which was stable for the rest of the observations. The patient was hemodynamically stable. Therefore, the consultant cardiologist did not pursue thrombolysis or catheter‐guided intervention.

## 4 Results and Conclusion

She was treated with Enoxaparin, which was then converted to Warfarin with Enoxaparin bridging after involving the hematology team. She improved symptomatically, was weaned off oxygen, and discharged. A follow‐up echocardiogram 3 months later demonstrated preserved biventricular size and systolic function without evidence of RV strain. A repeat ECG at the time shows the resolution of the ST changes. Although formal duplex ultrasonography of the lower limbs was not performed during the admission, the clinical suspicion for a lower extremity DVT as the embolic source was high, given the patient's recent immobility and prior history of PE. The decision not to pursue Doppler imaging was based on the definitive diagnosis of PE via CTPA and the absence of limb symptoms.

## 5 Discussion

### 5.1 ECG Findings in PE and Mechanisms of aVR Elevation

PE can present with various ECG changes, including ST‐segment elevation in lead aVR, which may mimic ACS [5]. It has been described in previous case reports [6]. A retrospective analysis identified ST elevation in lead aVR in approximately 34.3% of patients with acute PE, especially those with intermediate‐ to high‐risk features and signs of RV overload [5]. Similarly, another study documented transient ST elevation in lead aVR during hemodynamic collapse in patients with massive PE, suggesting that this finding may serve as a surrogate for acute RV ischemia [7]. These findings are important, as the same ECG pattern—ST elevation in aVR with widespread ST depressions—is also seen in critical left main or triple‐vessel coronary disease, leading to potential misdiagnosis and diversion toward unnecessary coronary angiography. The lack of specificity of this ECG pattern underscores the need for rapid bedside imaging, including echocardiography, and integration of clinical probability scores to avoid diagnostic delay. This phenomenon is particularly significant as it can lead to misdiagnosis and inappropriate treatment. ST‐segment elevation in aVR has been documented in several studies, indicating its potential as a marker for severe underlying conditions, including PE. Pathophysiology may involve increased RV wall tension, subendocardial ischemia, and reduced cardiac output, secondary to acute afterload mismatch. Similar cases in the literature have described patients with PE presenting with ST‐segment elevation in aVR alongside widespread ST depressions, closely mimicking acute left main or multivessel coronary disease [6, 7]. For instance, a report by Couto et al. described a patient with massive PE and aVR elevation treated initially as a STEMI, with normal coronary angiography and subsequent confirmation of PE [6]. Another study by Zhan et al. identified transient aVR elevation during hemodynamic collapse in patients with PE, correlating with right heart strain [7]. These cases, like ours, underscore the diagnostic dilemma posed by this ECG pattern and highlight the importance of bedside imaging and clinical integration to avoid delays in appropriate therapy.

### 5.2 Differential Diagnosis Challenges

This differentiation is critical, as misdiagnosis may lead to inappropriate intervention, such as unnecessary coronary angiography or delayed anticoagulation therapy. Our case exemplifies this dilemma, with the patient initially treated as a STEMI before the correct diagnosis was established.

Furthermore, in instances where a patent foramen ovale is present, paradoxical embolism may contribute to ST‐segment changes, complicating the clinical picture [8]. Case reports have documented PE presenting with ST‐segment elevation in multiple precordial leads, such as V1–V4, alongside typical signs of right heart strain, including the S1Q3T3 pattern and right axis deviation [9]. In our case, the patient was initially treated as a STEMI and taken for emergency coronary angiography, which revealed unobstructed coronaries. Bedside echocardiography at the catheter lab provided crucial diagnostic clarification by revealing RV dilatation, prompting CTPA and the correct diagnosis of pulmonary embolism. This case highlights the importance of integrating clinical, imaging, and laboratory findings rather than relying solely on ECG in chest pain evaluation. Table 1 explains the ECG differences between PE and ACS patterns.

**TABLE 1:** Key ECG differences between pulmonary embolism and acute coronary syndrome.

ECG feature	Pulmonary embolism (PE)	Acute coronary syndrome (ACS)
Sinus Tachycardia	Common	Less common
S1Q3T3 Pattern	Suggestive of PE (not always present)	Not characteristic
Right Axis Deviation	May be present	Rare
T‐wave Inversion in V1–V3	Suggestive of RV strain	Seen in Wellens syndrome (LAD), but usually in V2–V4
ST Elevation in aVR	Due to RV strain/ischemia	Common in left main or triple‐vessel disease
Diffuse ST Depression	Often accompanies aVR elevation	May indicate widespread subendocardial ischemia
RBBB or Incomplete RBBB	Frequently observed	Possible, but less frequent
Localized ST Elevation (e.g., II, III, aVF, or V1–V4)	Uncommon unless coexisting MI	Typical of STEMI depending on infarct location
Q waves	Rare	Common in established infarction

Abbreviations: ACS, acute coronary syndrome; LAD, left anterior descending artery; PE, pulmonary embolism; RBBB, right bundle branch block; RV, right ventricular; STEMI, ST elevation myocardial infarction.

## 6 Prognostic Implications

Recognising aVR changes in the context of PE may signify a critical condition requiring immediate intervention. Moreover, studies have shown that ST‐segment elevation in aVR serves as a poor prognostic marker in patients with PE, particularly in those classified as intermediate risk [5, 7, 10]. The predictive value of this ECG finding underscores the need for clinicians to maintain a high index of suspicion for PE in patients presenting with such ECG changes, especially when accompanied by clinical signs of right heart failure or hemodynamic instability [11, 12]. Some studies suggest that it could be incorporated into risk stratification models to identify high‐risk PE patients who require more aggressive intervention [11]. Further research is needed to establish whether this ECG finding should influence clinical decision‐making, particularly in normotensive patients with RV dysfunction.

## 7 Conclusion

ST‐segment elevation in lead aVR is a rare but important ECG finding in patients with PE, potentially leading to diagnostic confusion with ACS. This case emphasizes the need for heightened clinical suspicion and comprehensive assessment in patients with atypical ECG findings. As emerging evidence suggests a link between aVR elevation and adverse outcomes in PE, further research is warranted to determine its role in risk stratification and clinical decision‐making. Recognizing this presentation may also prompt early consideration of alternative diagnoses such as PE within STEMI protocols, especially when coronary angiography is unrevealing.

## Author Contributions

**Bernard R. Francis:** resources, writing – review and editing. **Nouman Arshad:** resources, writing – review and editing. **Mohammad El‐Din:** writing – review and editing. **Ibrahim Antoun:** conceptualization, writing – original draft.

## Ethics Statement

The authors confirm that written consent was obtained before submission of the case report.

## Consent

The patient has provided informed consent for publication.

## Conflicts of Interest

The authors declare no conflicts of interest.

## Data Availability

Data regarding this case report is available upon request to the corresponding author.
